# Feasibility, satisfaction, and preliminary results of a coping skills training program for parents of children with cancer

**DOI:** 10.1093/jpepsy/jsag015

**Published:** 2026-03-02

**Authors:** Oz Hamtzani, Michael J Dolgin, Talma Kushnir

**Affiliations:** Department of Psychology, Ariel University, Ariel, Israel; The Edmond and Lily Safra Children’s Hospital, Sheba Medical Center, Ramat Gan, Israel; Department of Psychology, Ariel University, Ariel, Israel; Department of Psychology, Ariel University, Ariel, Israel; The Adelson School of Medicine, Ariel University, Ariel, Israel

**Keywords:** oncology, coping skills and adjustment, stress, pilot/feasibility trial, caregiver functioning

## Abstract

**Objective:**

While parents of children with cancer experience increased levels of emotional distress, considerable variability in adjustment and distress is evident. The current study aimed to examine the feasibility, acceptability, and satisfaction of a semi-structured intervention protocol based on a three-factor theoretical model of coping and distress using a single-arm study design. The intervention focused on reducing the use of avoidance-focused coping techniques, expanding the repertoire of problem-, and emotion-focused coping techniques, and increasing flexibility in applying these techniques.

**Methods:**

A sample of 22 mothers and 10 fathers (*N *= 32) of children undergoing active cancer treatment were recruited from a pediatric hematology-oncology department. The manualized intervention protocol consisted of six 1-hr sessions. Parents completed standardized measures at three time points: baseline, post-intervention, and 6-week follow-up. Parents also completed an acceptability measure and four brief open-ended questions evaluating their satisfaction and experience with the intervention.

**Results:**

Of 48 eligible parents who were approached, 32 agreed to participate in the intervention (enrollment rate = 66.67%) and of these, 25 completed the intervention (retention rate = 78.13%), supporting the intervention’s feasibility. Parents who completed the intervention provided positive feedback and high satisfaction ratings. Descriptive patterns across pre-, post-, and follow-up assessments reflected expected directions of change in coping and distress measures.

**Conclusions:**

These results support the feasibility and acceptability of a theory-driven intervention based on a three-dimensional model of parental coping and distress. Although preliminary descriptive changes were observed across assessment points, these should not be interpreted as evidence of efficacy. Larger controlled trials are necessary to rigorously evaluate the intervention’s effectiveness.

Extensive research has documented the profound psychological burden experienced by parents whose children are diagnosed with cancer. Facing the complex challenge of preserving both individual and family functioning while simultaneously navigating medical care and intensive treatment protocols, caregivers experience elevated rates of anxiety, depression, and posttraumatic stress symptoms, as consistently demonstrated in the literature (Bakula et al., 2020; Borrescio-Higa & Valdés, 2022; Srivastava et al., 2020). In response to these mental health challenges, research efforts have turned to developing psychotherapeutic interventions for improving coping and decreasing parental emotional distress. Coping is defined as efforts to prevent or diminish threat, harm, and loss or to reduce the distress that is often associated with stressful experiences (Carver, 2020).

## Coping

Coping techniques emerge from individually selected coping strategies, organized patterns of actions, cognitions, and behaviors in response to challenging circumstances (Carver, 2020). Three primary coping strategies are commonly referred to: problem-focused, emotion-focused, and avoidance-focused strategies (Endler & Parker, 1994; Folkman, 2020). Empirical findings demonstrate that when stressors are modifiable, problem-focused approaches yield better psychological adjustment, whereas emotion-focused strategies prove more beneficial when stressors remain unalterable (Carver, 2020; Folkman, 2020). Based on the person’s choice of coping strategy, one may employ specific coping techniques. Problem-focused coping techniques involve acting on the environment (e.g., seeking instrumental support from others such as seeking practical help with daily tasks) or on the self (e.g., planning, active coping) to affect and change the nature of stressful situations. Emotion-focused coping techniques aim to regulate one’s emotions (e.g., prayer, relaxation, or seeking emotional support from others such as talking with a friend to share concerns and gain emotional validation) when the situation cannot be altered. Regardless of stressor characteristics, coping techniques oriented toward avoidance (e.g., denial) or self-attribution of blame typically correlate with elevated distress (Akbari et al., 2022).

While the strategy- and technique-based framework has been central to the coping literature, it does not fully encompass all theoretical approaches to understanding coping and distress. The literature has also conceptualized coping as involving broader processes such as meaning-making (Park, 2010; Park & Folkman, 1997) and benefit finding (Cassidy et al., 2014), particularly in the context of severe and ongoing stressors such as childhood cancer. Together, these perspectives underscore that no single theoretical framework fully captures the complexity of parental distress due to childhood cancer.

Despite theoretical diversity, studies suggest that no single coping technique consistently reduces distress across diverse stressors. Instead, the optimal coping process requires the ability to change and adjust over time according to the demands of the specific stressor and available resources (Lazarus & Folkman, 1984). This suggests that individuals possessing a broader repertoire of problem- and emotion-focused coping techniques can select contextually appropriate techniques for particular stressors, potentially achieving reduced emotional distress compared to those utilizing more limited repertoire, with possibly suboptimal coping techniques (Al‐Dajani et al., 2022; Golfenshtein et al., 2025; Heffer & Willoughby, 2017). Adaptive coping also requires flexible implementation of these techniques as a crucial mediating factor (Bonanno & Burton, 2013). Coping flexibility, defined as the capacity to abandon ineffective coping techniques and devise and implement alternative ones, has been identified as a pivotal factor in reducing emotional distress (Kato, 2020). Studies demonstrate that enhanced coping flexibility correlates with better emotional distress outcomes across various parent populations (Golfenshtein et al., 2025; Taubman Ben-Ari et al., 2024). Therefore, coping repertoire constitutes a fundamental prerequisite for flexibility, since adaptive flexibility cannot emerge without a variety of techniques available for selection (Bonanno & Burton, 2013). Recent cross-sectional evidence supports this framework, demonstrating that reduced reliance on avoidance strategy, broader repertoire of emotion- and problem-focused coping techniques, and a greater flexibility in applying these techniques, is significantly related to reduced parental distress levels in response to childhood cancer (Hamtzani et al., 2025).

## Interventions for parents of children with cancer

Studies suggest that parents of children diagnosed with cancer benefit from psychotherapeutic interventions. Recent systematic reviews and research have identified several key psychotherapeutic approaches in addressing parental distress due to childhood cancer, all of which have been rigorously evaluated and proven to be valid, reliable, and evidence-based in improving parental distress with effect sizes ranging from .20 to .70. These specific interventions are broadly based on problem-solving therapy (PST), cognitive-behavioral therapy (CBT), family-based therapy, resilience training programs, psychoeducation, and peer group support (Eche et al., 2021; Koumarianou et al., 2021; Luo et al., 2021; Tang et al., 2020; Zhou et al., 2024). Each of these interventions focuses on improving one or several specific coping techniques.

Bright IDEAS, a program based on PST (Nezu et al., 2012), introduced problem-solving skills training and aims at training parents to enhance their use of the *planning* technique by teaching parents a five-step problem-solving method (Identify the problem, Define available options, Evaluate those options and pick the best solution, Act and try to carry out the chosen solution, and See if it works). This intervention is applicable to planning solutions to situations parents commonly encounter during their child’s cancer treatment period, effective in reducing parental distress (Sahler et al., 2005, 2013; Zhou et al., 2024) with an effect size of approximately .50 (Dolgin et al., 2021).

Another well-established psychotherapeutic intervention is the Surviving Cancer Competently Intervention Program for parents of Newly Diagnosed children with cancer (SCCIP-ND; Kazak et al., 2005). The intervention is grounded in an integration of cognitive-behavioral, and family-based therapeutic approaches. The intervention is delivered in three structured sessions and is guided by four core components: establishing a collaborative therapeutic alliance with the family, maintaining an interpersonal focus, normalizing family experiences in the context of pediatric cancer, and strengthening family resources, resilience, and growth (Canter et al., 2019; Kazak et al., 1999, 2005). Within this integrated framework, CBT components aim to help parents identify and restructure maladaptive cognitions related to the child’s illness (e.g., positive reframing technique) and to increase engagement in manageable and rewarding activities (e.g., active coping, humor, religion or emotional-support techniques), thereby reducing individual distress. In parallel, family-based components focus on enhancing communication, mutual support, and shared coping within the family system, addressing relational processes that influence parental distress (Eche et al., 2021; Koumarianou et al., 2021; Tang et al., 2020).

Resilience training programs aim to enhance parents’ capacity for emotional self-regulation and adaptive cognitive responses. Techniques include mindfulness exercises, stress reduction, and social support enhancement to help parents maintain well-being over time, despite the ongoing stress of caregiving (Luo et al., 2021). Psychoeducational approaches provide parents with critical information about their child’s illness, treatment, and coping, aiming to decrease uncertainty and improve caregiver confidence (Koumarianou et al., 2021; Tang et al., 2020). Finally, peer groups provide social support through shared experiences and emotional expression to help alleviate isolation and reduce emotional burden (Koumarianou et al., 2021). These interventions often create a community context where parents can find validation and mutual understanding.

Despite these well-documented benefits, these interventions are designed to strengthen a limited set of specific coping techniques within clearly defined therapeutic frameworks. This observation highlights the potential value of interventions that integrate multiple coping techniques within a more flexible and holistic framework, allowing parents to draw on a broader repertoire of techniques and adapt their coping to diverse and evolving stressors.

## Current study rationale and objectives

The study aims to address this gap through the development of a comprehensive coping skills training program that integrates the three core coping constructs. The objectives of this program are to (1) reduce reliance on avoidance-focused coping strategies, (2) expand the repertoire of both problem-, and emotion-focused coping techniques, and (3) enhance flexibility in the application of these coping techniques. A brief, semi-structured intervention was developed and tested using a single-arm design among parents of children diagnosed with cancer. The aims of the current study were to evaluate: (1) feasibility outcomes including enrollment (parents’ consent to participate) and retention (number of parents who completed the intervention); (2) participant satisfaction and feedback; and (3) preliminary indications of efficacy.

## Methods

### Participants

Participants were recruited from the population of parents of children diagnosed with cancer in the pediatric hematology-oncology department of the Edmond and Lily Safra Children’s Hospital in Israel between April 2022 and May 2023. In the current study, the term “parents” refers broadly to the child’s primary caregivers, including biological parents as well as adoptive parents or other primary familial caregivers involved in the child’s daily care. Parents of children with any form of malignancy or central nervous system tumor and during all phases of active treatment were offered participation. Further eligibility criteria included the ability to speak and read Hebrew or English. Parents were approached no earlier than 6 weeks post-diagnosis in order to avoid potential burden during the initial period following diagnosis and treatment initiation. Additionally, parents whose child was in an acute medical crisis or was diagnosed with a terminal disease during the recruitment phase were not approached for participation. As part of routine care in the department, all families received standard psychosocial services, including social worker support, educational services, and psychological care for the child, with parental guidance as needed. Although receipt of additional psychosocial or mental health support for parents was not an exclusion criterion and not formally assessed, none of the parents reported receiving such support outside the hospital’s standard care.

The current study determined the required sample size based on the “traffic light” framework criteria (Lewis et al., 2021). The calculation is based on statistical power (beta), type I error rate (alpha), and two predetermined cut-offs for feasibility estimation (an upper and a lower limit, which will be detailed in the Measures section). To achieve approximately 95% power for each of two feasibility measures (i.e., study enrollment and retention), ensuring a collective power of about 90% (0.95^2^), with a type I error rate of 5%, a sample size of 31 participants was required. The current study successfully recruited 32 participants.

### Intervention

#### Protocol development

The intervention consisted of six 60-min individual sessions delivered by the first author. Sessions were offered in face-to-face, remote, or hybrid formats, based on parents’ preferences. Although parents were encouraged to attend sessions on a weekly basis, scheduling remained flexible to accommodate medical demands, hospitalizations, and parent availability. Sessions were conducted in both outpatient and inpatient settings. Three materials were developed: a therapist manual, an intervention PowerPoint, and parent worksheets (protocol and materials are available upon request). These materials were developed by the authors and revised by parents during beta-testing. The intervention was primarily informed by cognitive-behavioral and problem-solving therapies. Core components included establishing positive problem orientation, problem definition process, reducing the use of avoidance-focused coping techniques, expanding repertoire of coping techniques, and enhancing flexibility in applying these techniques, in order to reduce parental distress.

Fidelity was monitored in weekly supervision sessions. The intervention was administered face-to-face or remotely, based on the parent’s preference. The goals and intervention components are described in Table 1. Sessions were further informed by baseline questionnaires described in the “Measures” section.

**Table 1.** Outline of the intervention sessions.

Training session	Content
Training Session 1	1. Review of general intervention guidelines and goals: a. Establish a positive with the parent. b. Review intervention process and flowsheet. c. Establish positive expectancy.
Training Session 2	1. Introduce and discuss **cognitive problem orientation: positive vs. negative**. 2. Establish positive cognitive problem orientation: a. Illustrative vignette and discussion. b. Focus on the parent—discussion of the parent cognitive problem orientation. 3. Learn problem definition steps and the difference between **problem-focused and emotion-focused coping strategies**: a. Illustrative vignette and discussion. b. Focus on the parent—discussion of the parent’s current problems and selection of problem-focused or emotion-focused coping strategy.
Training Session 3	1. Learn and understand **problem-focused, emotion-focused, and avoidance-focused coping techniques**: a. Illustrative vignette and discussion. b. Focus on the parent—focus on a current problem the parent is facing, apply problem definition steps, choose a coping strategy and coping techniques, c. Evaluation of the process and discussion about the anticipated results.
Training Session 4	1. Continue to learn problem-focused and emotion-focused coping techniques, **focus on expanding coping techniques repertoire**: a. Illustrative vignette and discussion—problem-focused and emotion-focused coping techniques, use of multiple coping techniques. b. Focus on the parent—Continue to focus on the current problems of the parent’s choice.
Training Session 5	1. **Coping flexibility**—emphasize outcomes evaluation, abandonment, re-coping, and meta-coping processes: a. Illustrative vignette and discussion—coping flexibility. b. Focus on the parent—address the parent’s coping flexibility, continue to focus on the current problems of the parent’s choice.
Training Session 6	1. Review and consolidation of Sessions 1–5. 2. Focus on the parent: a. Continue to focus on current problems of the participant’s choice, problem definition steps, choice of problem-focused or emotion-focused strategy, choice of coping technique, abandonment, re-coping, and meta-coping. 3. Wrap-up. 4. Administer T2 assessment and schedule T3 assessment.

*Note:* Key coping constructs addressed in each session are highlighted in bold.

**Session 1.** The first session focused on establishing therapeutic rapport, clarifying the goals and structure of the intervention, and addressing parents’ questions and expectations. Parents were invited to share information about their family context, their child’s diagnosis, coping resources, and experience so far. The intervention was introduced as a coping skills training, aimed at improving coping with the challenging demands relevant to parents.

**Session 2.** The second session focused on developing a positive problem orientation and a structured problem definition process. Parents were guided to systematically identify and break down challenges into manageable components and to distinguish between stressors that could be altered (which later will require a problem-focused coping technique) or not (which will require an emotion-focused coping technique). Emphasis was placed on the subjective and dynamic nature of this appraisal, which may change over time based on available coping resources.

**Session 3.** The third session focused on increasing parents’ awareness of common avoidance, emotion-focused, and problem-focused coping techniques used in response to stress. The session emphasized the maladaptive role of avoidance-focused coping techniques in parental distress and encouraged greater reliance on emotion- and problem-focused coping techniques.

**Session 4.** The fourth session emphasized the importance of a broad repertoire of emotion- and problem-focused coping techniques. Parents were encouraged to generate multiple potential coping techniques for different challenges and to develop a structured action plan, detailing implementation steps, timing and involving parties.

**Session 5.** The fifth session focused on coping flexibility, highlighting three core components (Kato, 2020): abandonment (i.e., the ability to relinquish a coping technique recognized as ineffective), re-coping (i.e., the ability to implement another appropriate technique), and meta-coping (i.e., the ability to monitor and to provide feedback on the effect of the coping process). Parents were guided to flexibly revisit earlier sessions’ steps when selected coping techniques were subjectively insufficient.

**Session 6.** The sixth and final session focused on reviewing and integrating the coping skills and processes introduced throughout the intervention. Parents provided qualitative feedback and completed post-intervention outcome and satisfaction measures.

#### Beta testing

Beta testing was conducted on the first three participants as a formative process in order to refine the intervention prior to full implementation. Based on parents’ feedback, problem orientation was postponed from Session 1 to Session 2 to allow additional time for establishing rapport with the parent. In addition, the intervention initially included homework assignments to facilitate coping skills practice. However, this was relaxed following parents’ feedback indicating difficulties with consistent completion due to caregiving demands. Parents’ most common everyday concerns were solicited to ensure that the intervention addressed issues most relevant to their experiences and to inform the development of the clinical vignettes incorporated into the intervention. The beta-testing phase was considered complete once no additional procedural or content-related modifications were identified.

### Measures

#### Demographic and medical data

Parents were asked to provide demographic and background information, including gender, age, place of birth, education level, marital status, number of children, cancer history (i.e., whether the parent had a first-degree family member with a past or current cancer diagnosis, included to account for prior exposure to cancer-related stress that may influence parental distress and coping; Hamtzani et al., 2025), and religious affiliation. In addition, parents provided information regarding their child, including gender, age, diagnosis, time since diagnosis, and treatment protocol.

#### Intervention feasibility

According to the “traffic light” system (Lewis et al., 2021), feasibility metrics of enrollment and retention are classified as “GREEN” (feasible), “AMBER” (needs modification), or “RED” (substantial issues), with overall progression determined by the worst-performing criterion. Psychological intervention programs for parents of children with cancer face multiple barriers related to recruitment and retention, as parents often report difficulties focusing on anything beyond their child’s immediate treatment needs (Canter et al., 2020; Hocking et al., 2014). Based on these data, we established the enrollment metric boundaries at 35%–65% and retention at 40%–70%.

#### Brief COPE

Coping techniques were assessed using the Hebrew version of the Brief COPE (Ben-Zur & Zeidner, 1995) originally developed by Carver (1997). This 28-item self-report questionnaire assesses the utilization of 14 coping techniques rated on a 0–4 scale from “I haven’t been doing this at all” to “I’ve been doing this a lot”. Scoring is based on the average of the two items that make up each coping technique. Parents were asked to report on coping techniques used specifically in response to their child’s illness. From this instrument, we derived measures of avoidance-focused coping strategy and coping repertoire as described below.

**Coping strategies.** Following Endler & Parker’s (1994) classification, the 14 coping techniques were grouped into avoidance-focused (behavioral disengagement, denial, substance use, self-blame; α = .67, λ_2_ = .70), emotion-focused (self-distraction, venting, acceptance, humor, religion, positive reframing, emotional support; α = .70, λ_2_ = .73), and problem-focused (instrumental support, planning, active coping; α = .80, λ_2_ = .81) strategies. Religious coping was excluded from repertoire scoring due to methodological considerations (Hamtzani et al., 2025).

**Coping repertoire.** Consistent with Heffer & Willoughby (2017), each coping technique was binary coded (0—“non-utilization”, 1—“any degree of utilization”) and summed to derive an individual coping repertoire score. Coping repertoire was operationalized as the total number of neutral-to-positive coping techniques endorsed. This classification was based on empirical correlations with parental distress in an independent study (Hamtzani et al., 2025), identifying techniques associated with neutral or reduced parental distress in childhood cancer. Neutral-to-positive coping techniques included acceptance, humor, positive reframing, social emotional support, social instrumental support, planning, and active coping, whereas techniques associated with negative outcomes included behavioral disengagement, denial, substance use, self-blame, self-distraction, and venting. Resulting coping repertoire scores ranged from 0 to 7, with higher scores indicating a broader neutral-to-positive coping repertoire.

#### Coping Flexibility Scale-Revised

The Coping Flexibility Scale-Revised (CFS-R) (Kato, 2020) includes 12 items that assess coping flexibility through three 4-item subscales: Abandonment (e.g., “I can stop using a failed coping technique”), Re-coping (e.g., “If I did not cope well, I use an alternative coping technique”), and Meta-coping (e.g., “I cope with stress by establishing clear objectives”). Items are rated on a four-point scale from 0 (“not applicable”) to 3 (“very applicable”), with greater scores reflecting greater flexibility. The instrument demonstrates strong psychometric properties across the three subscales: abandonment (Cronbach α = .82), re-coping (Cronbach α = .84), and meta-coping (Cronbach α = .80). For this research, the original English CFS-R was translated into Hebrew using translation and back-translation methodology. We computed a composite flexibility score using only the “Abandonment” and “Re-coping” subscales following Kato’s recommendations.

#### Pediatric Parenting Stress Inventory

The Hebrew version of the Pediatric Parenting Stress Inventory (PPSI) (Dolgin et al., 2018), originally developed by Devine et al. (2014), was used to assess parental distress. The PPSI is a 35-item self-report measure rated on a 0–4 scale from “not at all” to *to a “*very large extent”, reflecting the perceived severity of each problem during the preceding week. The instrument generates an overall stress score, alongside four domain-specific subscales reflecting common parental challenges: Managing child’s needs, Emotional and physical problems of the parent, Managing finances, and Managing family life. For the purposes of the current study, only the emotional and physical problem sub-scale was employed (Cronbach α = .94), which was found to correlate highly with overall mood, depression, and posttraumatic stress symptoms (Devine et al., 2014; Dolgin et al., 2018). Higher scores indicate greater parental distress.

#### Profile of Mood State Short Form

The Hebrew version of the Profile of Mood State Short Form (POMS-SF) (Netz et al., 2005), originally developed by Shacham (1983), was used to assess overall affective mood disturbance. The POMS-SF is a 28-item self-report measure designed to measure affective mood states experienced over the previous week in five dimensions: 1. Anger-Hostility; 2. Depression-Dejection; 3. Fatigue-Inertia; 4. Tension-Anxiety, and 5. Vigor-Activity. Items are rated on a 0–4 scale from “not at all” to *to “*a very large extent”. For the purposes of the current study, the general mood score was used (Cronbach α = .91). Higher scores reflect greater mood disturbance.

#### Treatment Acceptability Questionnaire

The Treatment Acceptability Questionnaire (TAQ) (Hunsley, 1992) is a 6-item self-report measure assessing intervention acceptability and satisfaction, rated on a 7-point Likert scale ranging from “Strongly disagree” to “Agree strongly”, with a higher score representing greater acceptance of the intervention. The *TAQ* was translated into Hebrew using translation and back-translation procedures, and items were adapted to refer specifically to the current intervention. Two additional items assessed parents’ willingness to repeat the intervention under similar circumstances and to recommend it to other parents.

#### Descriptive qualitative feedback

Parents who completed the intervention were asked to respond to four brief open-ended questions assessing their satisfaction and experience with the intervention. These questions addressed overall satisfaction, aspects of the intervention parents found most helpful, aspects they found less helpful, and suggestions for improvement. Feedback was provided either verbally during the sixth session or in written form, according to parents’ preference.

### Procedure and ethics

Measures were administered at baseline (T1—demographic and medical data, Brief COPE*,* CFS-R*,* PPSI, and POMS-SF), post-intervention (T2—Brief COPE*,* CFS-R*,* PPSI*,* POMS-SF, and TAQ), and at 6-week follow-up (T3—Brief COPE*,* CFS-R*,* PPSI, and POMS-SF). Eligible participants were asked to sign an informed consent that included a written description of the study, its goals and contribution, confidentiality, risks, and benefits, and the right to withdraw from the study at any given time. The study was approved by the ethics committees (Helsinki) of both Ariel University (AU-SOC-TK-20220315) and the Edmond and Lily Safra Children’s Hospital (SMC-9041-22). We followed the CONSORT 2010 checklist criteria for reporting this feasibility study (Eldridge et al., 2016). The checklist is available as Supplementary File S1.

### Data analyses

Given the feasibility and acceptability focus of the present study, analyses were primarily descriptive. Feasibility outcomes were operationalized as enrollment and retention rates and summarized using percentages. Acceptability and satisfaction were assessed using mean scores from the TAQ as well as a descriptive review of open-ended feedback. Open-ended responses were reviewed by the research team to identify recurring topics and illustrative examples related to intervention acceptability. Specifically, the authors reviewed the entire set of responses to generate a summary of common feedback points. These observations were then discussed jointly to ensure the final summary accurately captured the range of parents’ perspectives. Importantly, this feedback was not subjected to formal qualitative analysis (e.g., systematic coding, multiple independent coders, or thematic saturation) and is therefore presented summarily to inform feasibility and acceptability rather than to generate qualitative themes.

For exploratory quantitative outcomes, means and standard deviations were calculated for all self-report measures at baseline, post-intervention, and 6-week follow-up. Consistent with recommendations for feasibility studies, no inferential statistical analyses or formal hypothesis testing were conducted. In addition, effect sizes were not calculated since in small samples they can be unstable and may over-, or underestimate the true magnitude of effects (Teresi et al., 2022).

## Results

### Intervention feasibility

**Figure 1.**
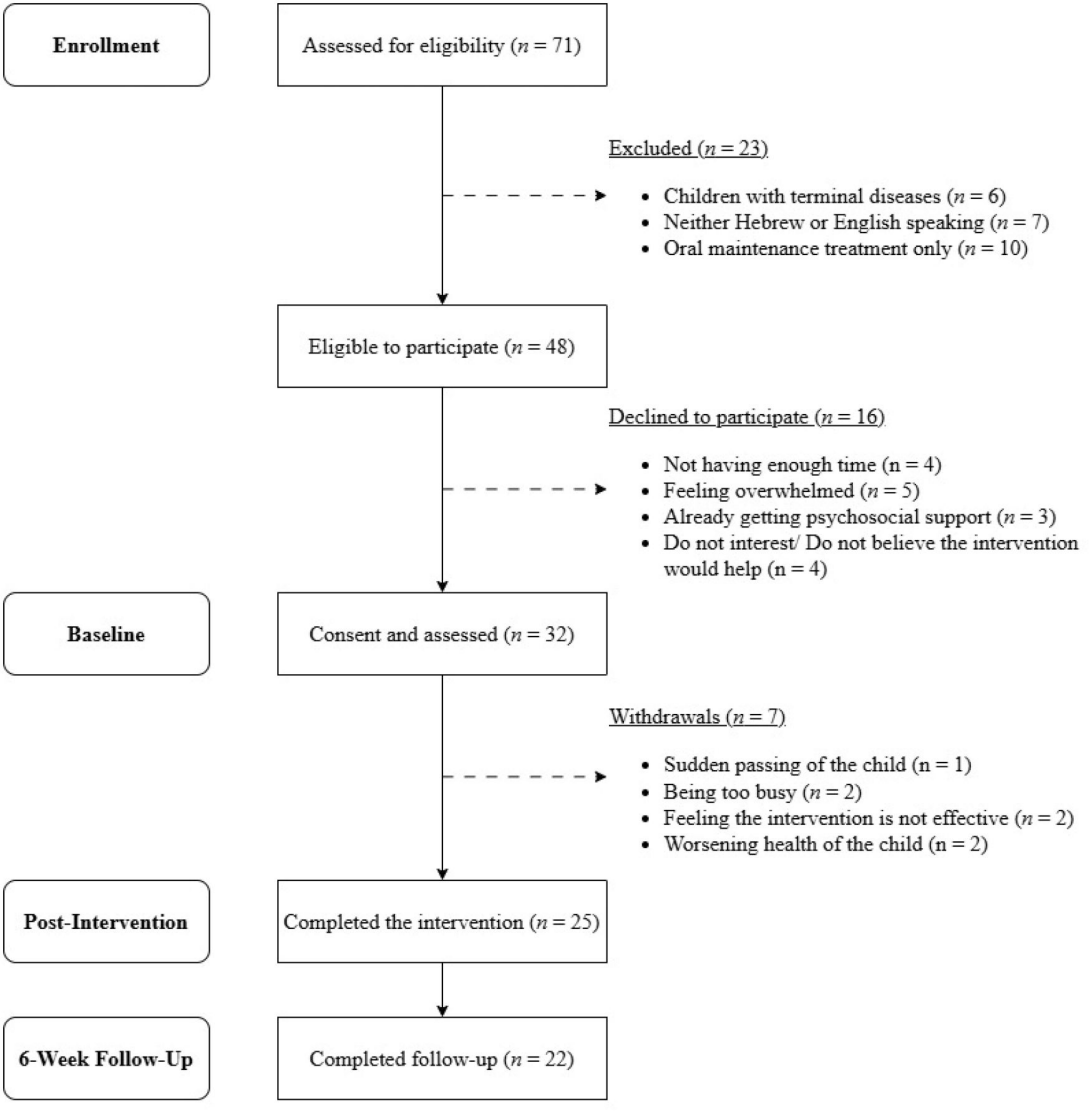
CONSORT flow diagram.

Figure 1 presents the CONSORT flow diagram. Seventy-one parents were assessed for eligibility to participate in the study. Of the 48 eligible participants, 32 parents consented to participate (66.67%). There were no differences between participants and non-participants regarding parent/child demographic or medical characteristics, with the exception that the majority of parents who refused to participate were ultra-orthodox (Haredim) (61%, *χ*^2^(3) = 19.35, *p* < .001).

Of the 32 parents enrolled, 25 (78.13%) completed all six sessions. Seven participants withdrew: three after the first session and four after four sessions. Specific withdrawal reasons are detailed in Figure 1. Twenty-three of the participants chose to receive the intervention in face-to-face format, and five of them withdrew. Two of the participants who received the intervention remotely withdrew from the intervention. All the participants who received the intervention in a hybrid format completed all the sessions. The current study’s enrollment (>65%) and retention (>70%) rates fall within the green zone (Lewis et al., 2021), indicating acceptable feasibility. Sessions were completed over an average of 8.46 weeks (SD = 3.58).

### Participants

Table 2 displays parental demographic and child medical data. The intervention sample included 69% mothers and 31% fathers, with ages ranging from 26 to 52 years. All participants were Hebrew-speaking, and therefore all assessments were administered in Hebrew. The majority of parents were married or in a relationship, and had a bachelor’s degree or other professional certification. All participants were Jewish, with 50% identified themselves as secular (i.e., not practicing religion). Only one participant was ultra-orthodox, and no significant differences were found among participants in cancer history, income level, or number of children. The majority of parents chose the face-to-face intervention platform, while others preferred remotely only or hybrid. Finally, most children were newly diagnosed, whereas only three were being treated for recurrent disease. Finally, two parents were a divorced couple. We included both in the analyses, given the feasibility nature of the study, and because they had been divorced for several years, resided in separate households, and participated independently.

**Table 2.** Parent demographics and child medical characteristics (*N* = 32).

Measure		Statistical test
Parents characteristics		
Gender*, n (%)*		
Mothers	22 (69%)	χ²(1) = 4.50, *p* = .03
Fathers	10 (31%)	
Age, *M* (*SD*)	41.38 (7.99)	
Marital status*, n (%)*		
Married/in a relationship	26 (81%)	χ²(1) = 12.50, *p* < .001
Not in a relationship	6 (19%)	
Level of religiosity*, n (%)*		
Secular	16 (50%)	χ²(3) = 15.75, *p* < .001
Traditional	5 (16%)	
Orthodox	10 (31%)	
Ultra-orthodox	1 (3%)	
Race*, n* (%)		
Ashkenazi Jewish	16 (50%)	χ²(2) = 10.75, *p* = .01
Mizrahi/Sephardic Jewish	14 (44%)	
Other	2 (6%)	
Cancer history*, n* (%)		
No	15 (47%)	χ²(1) = .13, *p* = .72
Yes	17 (53%)	
Education*, n* (%)		
High school education	4 (13%)	χ²(3) = 11.25, *p* = .01
High school diploma	5 (16%)	
Bachelor’s degree/professional certificate	16 (50%)	
Master’s degree or higher	7 (22%)	
Level of income*, n* (%)		
Under average national income	15 (47%)	χ²(2) = 2.69, *p* = .26
Average national income	9 (28%)	
Above national income	8 (25%)	
Number of children*, n* (%)		
1	4 (13%)	χ²(3) = 3.25, *p* = .35
2	8 (25%)	
3	9 (28%)	
4+	11 (34%)	
Intervention platform, *n* (%)		
Face to face	23 (72%)	χ²(2) = 21.44, *p* < .001
Remotely	4 (13%)	
Hybrid	5 (16%)	
Child’s medical characteristics		
Gender*, n* (%)		
Female	13 (41%)	χ²(1) = 1.13, *p* = .29
Male	19 (59%)	
Age, *M* (*SD*)	8.41 (5.26)	
Diagnosis*, n* (%)		
CNS	13 (41%)	χ²(4) = 15.50, *p* < .001
Leukemia	5 (16%)	
Lymphoma	1 (3%)	
Sarcoma	3 (9%)	
Solid tumor	10 (31%)	
Child’s position*, n* (%)		
1	13 (41%)	χ²(3) = 5.50, *p* = .14
2	9 (28%)	
3	5 (16%)	
4+	5 (16%)	
Weeks since diagnosis, *M* (*SD*)	12.85 (4.39)	
Initial vs. relapsed disease*, n* (%)		
Initial	29 (91%)	χ²(1) = 21.13, *p* < .001
Relapsed	3 (9%)	

Children showed similar gender proportions, with ages ranging from 0 to 17 years. The majority had central nervous system (CNS) or solid tumor diagnoses, received chemotherapy, and half received radiation therapy. The intervention did not include parents of children who had undergone bone marrow transplant due to isolation requirements of the hospital during the COVID epidemic.

### Participant satisfaction and feedback

The TAQ was administered at post-intervention (*N* = 25). Parents who completed the intervention (*N *= 25) reported high satisfaction ratings (on a 7-point Likert scale) with the intervention overall (*M* = 6.36, *SD* = .65), including items addressing intervention acceptability, ethics, and effectiveness. No difference in satisfaction was found between parents who received the intervention face-to-face (*M* = 6.46, *SD* = 0.51) and those who received the intervention remotely or in a hybrid format (*M* = 6.14, *SD* = 0.89); (*t* (9.20) = 0.92, *p* = .38).

A descriptive summary of parents’ open-ended feedback illustrates the intervention’s acceptability and identifies opportunities for future refinement. Many noted it helped them refine existing coping techniques to better manage daily challenges. The brief, flexible format (in-person or remote) facilitated engagement, while materials and vignettes were praised for supporting reflection and problem-solving. However, most parents felt the intervention was too short, suggesting an extension to eight sessions or adding follow-ups. Recommendations also included tailoring vignettes to specific developmental stages and family contexts (e.g., infants vs. older children). Table 3 shows examples of the feedback provided.

**Table 3.** Illustrative parents feedback regarding intervention acceptability and satisfaction.

Category	Illustrative quotes
Overall	“I feel like I knew the majority of it, but during that stressful time, you reminded me that I know it and that I can use it”
	“It is a good thing because it’s kind of gives you hope that you can cope with the situation”
What did you like the most about the intervention?	“The vignettes were good and give some good examples of what we parents have to deal with. The fact that we used it to improve our coping made it even better”
	“The worksheets helped me a lot. Lately I feel that I always have to think fast and you kind of made me stop for a second, write it down and think”
What did you not like about the intervention?	“It kind of feels robotic sometimes, I don’t know If I can always use it like we did it”
	“I felt like the vignettes were not age appropriate. I’m sure there are parents who felt the vignettes were extremely helpful, but my daughter is 7 months old, and it just doesn’t seem fit”
How can the intervention be improved?	“Add more sessions. On the one hand, we parents are busy, and we do need something short and to the point. On the other hand, after six sessions I only started to get it so maybe another 2-4 more sessions would be better, but no more than that”
	“Make a follow-up session. Just one session, like a month after the end of the intervention just to catch-up and see if any adjustments are mended”

### Secondary results

Given the relatively small sample sizes of feasibility studies, the preliminary results on intervention effects are presented descriptively without formal hypothesis testing, serving as an initial indication rather than definitive evidence of efficacy (Teresi et al., 2022). Twenty-five parents completed both baseline and post-intervention questionnaires, while 22 also completed the 6-week follow-up questionnaires.

Overall, parents showed changes in the expected directions across primary (reduced use of avoidance-focused coping strategy, broader coping repertoire of techniques, and greater flexibility) and secondary (reduced parental distress) outcomes, as illustrated in Table 4.

**Table 4.** Means and standard deviations for outcome measures across study time points.

Measure	T1 (*n* = 32)	T2 (*n* = 25)	T3 (*n* = 22)
	*M* (*SD*)	*M* (*SD*)	*M* (*SD*)
Avoidance-focused coping strategy	1.09 (0.65)	0.89 (0.44)	0.93 (0.40)
Coping repertoire	6.66 (0.94)	7.04 (0.96)	7.04 (0.71)
Coping flexibility	1.95 (0.54)	2.17 (0.45)	2.18 (0.49)
PPSI emotional and physical problems	1.65 (0.87)	1.40 (0.83)	1.43 (0.81)
POMS total	1.73 (0.77)	1.48 (0.71)	1.47 (0.83)

## Discussion

The results of the current study support the intervention’s feasibility and acceptability among parents of children diagnosed with cancer. Both enrollment (i.e., 66.67% of parents who were approached agreed to participate in the intervention) and retention (i.e., 78.13% of parents who started the intervention completed it) fell within the “green zone” (Lewis et al., 2021), indicating that the predefined enrollment and retention benchmarks for the intervention were met (above 65% for enrollment and 70% for retention). The majority of parents reported that the intervention was easy to manage and practical to integrate into their daily routines, a finding that further supports its acceptability in the context of ongoing pediatric cancer treatment. Although overall satisfaction was high, parents also offered constructive suggestions for refinement. Most commonly, parents suggested extending the intervention by 1–2 additional sessions in order to further reinforce and consolidate the coping techniques introduced. Importantly, this feedback appears to reflect a desire for continued engagement and skill strengthening rather than dissatisfaction with the intervention.

While most of the parents chose the face-to-face delivery, feasibility and satisfaction did not differ across delivery formats. Notably, the majority of participants who declined participation were Ultra-Orthodox Jewish parents, a population that relies heavily on intra-community resources and may face cultural barriers to engagement in psychological interventions (Band-Winterstein & Freund, 2015; Doron et al., 2024).

Beyond Ultra-Orthodox communities, several populations in Israel remain underrepresented in mental health research and services, including individuals of Ethiopian origin (Shapiro et al., 2024), the Arab population (Daeem et al., 2019; Khatib et al., 2024), Circassian communities (Haron et al., 2004), and the Druze community (Ryder et al., 2021; Toukhy et al., 2022). These groups may encounter cultural and structural barriers to accessing psychosocial interventions. Future research should examine how the current intervention can be culturally adapted to enhance accessibility and acceptability across diverse and underrepresented populations. A strength of the current study is the relatively high representation of fathers (31%), which is notable given their frequent underrepresentation in pediatric psychology research and enhances generalizability across genders. Finally, descriptive patterns of coping and distress measures across three assessment points reflect the expected direction of change.

### Study limitations

The current study demonstrated the feasibility and acceptability of a manualized intervention protocol, which together provide a solid foundation for future randomized controlled trials (RCT). However, several limitations warrant consideration.

Most notably, the absence of a control group limits the ability to draw conclusions regarding the intervention’s effectiveness. Without a comparison to a non-intervention, waitlist, or alternative intervention group, observed changes cannot be attributed to the intervention, but rather to time, attention, and other non-specific factors. Additionally, the small sample size, inherent in feasibility studies, reduces statistical power and generalizability. Therefore, findings should be interpreted as preliminary and exploratory.

Second, the intervention emphasized expanding parents’ repertoire of coping techniques rather than training a limited number of specific ones. While this approach may enhance flexibility and address the heterogeneity of stressors faced by parents of children with cancer, it may be less optimal for some parents who benefit more from focused training and mastery of specific coping techniques.

Third, satisfaction and feedback were assessed only among parents who completed the intervention, introducing a potential risk of selection bias. Although only two non-completers explicitly reported withdrawing due to dissatisfaction, other parents who did not complete the intervention may have held different views regarding its acceptability or satisfaction. Sample characteristics also warrant consideration. While leukemia and lymphoma are the most prevalent pediatric cancer diagnoses, the relatively small sample size may influenced the observed diagnostic distribution, resulting in a higher proportion of CNS and solid tumors. In larger samples, diagnostic patterns would be expected to more closely reflect those reported in the broader pediatric oncology literature.

An additional limitation relates to the use of translated self-report measures. The CFS-R was translated into Hebrew using a standard translation and back-translation procedure. Although widely used to ensure linguistic equivalence, this approach may not fully capture contextual or cultural nuances (Behr, 2017). To our knowledge, the CFS-R has not yet been formally validated in Hebrew. In addition, with the exception of the POMS-SF (Sahler et al., 2005, 2013) and the PPSI (Dolgin et al., 2018), the Hebrew version of the Brief COPE has not been specifically validated among parents of children with cancer. There is also a potential risk of a “teaching to the test” effect on parents’ responses, insofar as the intervention and assessment contents were closely related (Schwarz et al., 2020). Finally, the commonly used three-factor structure of the Brief COPE may not fully capture the complexity of the coping process (Solberg et al., 2022), as suggested by the internal consistency of the subscales.

To address these limitations, future studies should employ RCTs with larger and more demographically diverse samples, incorporating diverse and independent measures to evaluate coping and distress. These studies should also include long-term follow-ups, as well as alternative forms of assessing the skills acquired through the intervention, such as others’ reports (e.g., spouse), stress and coping diaries, or responses to hypothetical (e.g., vignette) situations that reflect coping techniques, repertoire, and flexibility. Future studies may further benefit from a more personalized approach, in which parents are supported either by expanding their coping repertoire or by strengthening specific coping techniques based on individual preferences.

### Theoretical and clinical implications

In line with our earlier cross-sectional findings (Hamtzani et al., 2025), the current intervention was guided by our three-dimensional coping theoretical framework, emphasizing reduced use of avoidance-focused coping techniques, broader repertoire of emotion- and problem-focused coping techniques, and greater flexibility in applying them. These findings support further investigation of this integrative framework.

Clinically, the current intervention extends existing approaches by adopting a broader general coping skills training. Rather than focusing on the acquisition of specific techniques, the intervention aimed to enhance parents’ overall coping skills by strengthening their repertoire of coping techniques and flexibility in applying them to real-life challenges.

## Conclusions

This study evaluated the feasibility and acceptability of a semi-structured intervention for parents of children undergoing cancer treatment, guided by a three-dimensional coping model. Recruitment and retention rates met predefined feasibility benchmarks, indicating that the intervention is practical and well accepted. Participant feedback informed minor refinements to the protocol, supporting the suitability of the intervention for further evaluation in a randomized controlled trial designed to examine efficacy and mechanisms of change.

## Supplementary Material

jsag015_Supplementary_Data

## Data Availability

The data that support the findings of this study are available from the corresponding author upon reasonable request.
